# Examining the Driving Factors of Urban Residential Carbon Intensity Using the LMDI Method: Evidence from China’s County-Level Cities

**DOI:** 10.3390/ijerph18083929

**Published:** 2021-04-08

**Authors:** Jincai Zhao, Qianqian Liu

**Affiliations:** 1 School of Business, Henan Normal University, Xinxiang 453007, Henan, China; zhaojincai1989@163.com; 2 School of Geography Science, Nanjing Normal University, Nanjing 210023, Jiangsu, China; 3 Jiangsu Center for Collaborative Innovation in Geographical Information Resource Development and Application, Nanjing 210023, Jiangsu, China

**Keywords:** carbon intensity, residential sector, urban expansion, LMDI, county level

## Abstract

Improving carbon efficiency and reducing carbon intensity are effective means of mitigating climate change. Carbon emissions due to urban residential energy consumption have increased significantly; however, there is a lack of research on urban residential carbon intensity. This paper examines the spatiotemporal variation of carbon intensity in the residential sector during 2001–2015, and then identifies the causes of the variation by utilizing the logarithmic mean Divisia index (LMDI) with the help of Microsoft Excel 2016 for 620 county-level cities in 30 Chinese provinces. The results show that high carbon intensity is mainly found in large cities, such as Beijing, Tianjin, and Shanghai. However, these cities showed a downward trend in carbon intensity. In terms of influencing factors, the energy consumption per capita, urban sprawl, and land demand are the three most influential factors in determining the changes in carbon intensity. The effect of energy consumption per capita mainly increases the carbon intensity, and its impact is higher in the municipal districts of provincial capital cities than in other types of cities. Similarly, the urban sprawl effect also promotes increases in carbon intensity, and a higher degree of influence appears in large cities. However, as urban expansion plateaus, the effect of urban sprawl decreases. The land-demand effect reduces the carbon intensity, and the degree of influence of the land-demand effect on carbon intensity is also clearly stronger in big cities. Our findings show that lowering the energy consumption per capita and optimizing the land-use structure are a reasonable direction of efforts, and the effects of differences in influencing factors should be paid more attention to reduce carbon intensity.

## 1. Introduction

Climate change has become the most significant global environmental problem [1]. There is no doubt that, with the vigorous overall development of the global economy, carbon emissions from burning fossil fuels accelerate the process of climate warming [2,3,4]. According to statistics from BP in 2019, the growth rate of energy-related carbon emissions reached a record level in 2018—2.1% since 2010. Although the growth of carbon emissions dropped to 0.5% in 2019, the average annual growth over 2018 and 2019 was greater than its 10-year average [5]. Clearly, all governments must strive to reduce their carbon emissions.

China, as the biggest developing country in terms of land area and population, has become the largest carbon emitter [6,7]. Consequently, it is imperative to reduce carbon emissions and move onto a more sustainable path. With unremitting efforts, the energy efficiency of China has been significantly improved since the reform and opening-up [8], and China’s energy intensity decreased by 66.79% during 1980–2003 [9]. However, there is still a long way to go in order to reach China’s emission-reduction target. The residential sector is one of the three largest energy-consuming sectors, and therefore, low-carbon consumption by resident families is key in vigorously promoting the development of a low-carbon economy [10,11]. Additionally, China has been experiencing rapid urbanization in recent decades. According to the national statistics, the urbanization rate reached 60.60% in 2019 from 36.22% in 2000 [12], and the rate is predicted to reach 65.5% in 2025 [13]. Large-scale rural–urban migration is a powerful factor in the increase in residential energy consumption, and carbon emissions from such consumption in urban areas have dramatically increased. Therefore, the reduction of carbon emissions from urban residential energy consumption is key in slowing the process of climate warming.

In the context of sustainable development, there have been numerous studies quantifying the effects of various influencing factors on energy consumption and carbon emissions, such as urbanization rate [14,15], urban compactness [16], consumption level [17], economic development level [18], energy price [19], and climate factors [20]. In fact, improving carbon efficiency is an important way to develop a low-carbon economy and achieve carbon-emission reduction targets [21]. Scholars have conducted many studies on carbon-emission efficiency [22,23,24,25,26]. However, there is still no unique, quantified description of carbon efficiency [27]. Broadly speaking, carbon efficiency is an indicator of financial or other beneficial outputs gained concerning carbon emissions [28]. The ratio of gross domestic product (GDP) to carbon emissions is also defined as carbon efficiency [29], and is easily obtained—in this sense, carbon intensity, as a ratio of CO_2_ emissions to GDP, is regarded as the reciprocal of carbon efficiency, and is commonly used to represent a country’s energy and environmental performance [30,31].

In order to promote the green economy, significant research on energy intensity and carbon intensity has been conducted at the national, provincial, and city levels (displayed in Table 1). In the residential sector, several factors affecting carbon intensity have been detected, such as the number of households [32], population [33], and consumption structure [34]. However, these studies mainly focused on the influences of socioeconomic factors. In fact, anthropogenic activity has greatly increased as a result of human societal development, reflected by the continuous expansion of built-up areas. The effects of urban sprawl and land-use change on carbon emissions during such development have attracted a great deal of attention. For example, Li et al. [35] showed that an increase in city sizes produces a rise in CO_2_ emissions. Dong et al. [36] revealed that there is an inverted U-shaped curve between land urbanization and carbon-emission intensity. However, only a few studies were conducted to quantify the effect of urban sprawl on residential carbon emissions and carbon intensity. As an example, Rong et al. [37] deduced that urban sprawl drove the increase in urban residential carbon emissions according to the spatial patterns. There was barely direct evidence showing the influence of urban sprawl on residential carbon intensity. Therefore, it is necessary to reveal the effect of urban sprawl on changes in carbon intensity. Decomposition models are extensively utilized in quantifying the influences of driving factors on carbon emissions. Among them, the logarithmic mean Divisia index (LMDI) model could completely decompose a variable into multiple factors and eliminate the residual errors [38,39]. A significant amount of the literature employs the LMDI method to disentangle energy consumption, carbon emissions, and energy/carbon intensity into several socioeconomic variables [40,41,42]. Nevertheless, factors related to the land-use change, such as urban sprawl, are hardly reflected in the LMDI method.

Although much research has been done to mitigate climate warming, there are still some shortcomings. Firstly, few studies have focused on carbon intensity in the residential sector. As the second-largest emitter, the residential sector is crucial to carbon-emission reduction. Secondly, in assessing the impact of land-use change on carbon emissions using econometric models, the problem of endogeneity is hard to avoid. Therefore, further work is needed to incorporate land-use change into the LMDI model. Thirdly, few studies have paid close attention to the driving factors of carbon intensity for county-level cities, i.e., the effects of such factors in cities at different economic levels. Consequently, in this paper, there are three objectives: (1) to use the complete decomposed LMDI model to identify the effects of urban sprawl on residential carbon intensity in urban areas; (2) to compare regional differences regarding influencing factors using decomposition analysis at the county level; (3) to conduct a comprehensive analysis of the urban sprawl effect for cities at different economic levels.

The rest of the paper is organized as follows: Section 2 describes the methodology and data. The main results are presented in Section 3. Section 4 discusses the results, and Section 5 presents some conclusions and policy suggestions.

## 2. Materials and Methods

### 2.1. Extended LMDI Model

The LMDI method is a complete index decomposition method that does not produce a residual term and that is suitable for exploring the influencing factors of carbon intensity [50]; it has been widely used in numerous studies [51,52]. In the residential sector, income is a beneficial output for residents, so we defined the carbon intensity as the ratio of carbon emissions to income. We decomposed the urban residential carbon intensity by using the LMDI model to extract the main driver with the help of Microsoft Excel 2016. Using the Kaya identity, carbon intensity can be decomposed into six factors as follows:

(1) $$CI=\sum_{i}\frac{C_{i}}{E_{i}}\cdot \frac{E_{i}}{E}\cdot \frac{E}{P}\cdot \frac{P}{B}\cdot \frac{B}{A}\cdot \frac{A}{I}=\sum_{i}CC_{i}\cdot ES\cdot EP\cdot PD\cdot URS\cdot LD$$

where *CI* is the carbon intensity in the residential sector—the smaller the value, the higher the carbon efficiency. *E_i_* and *C_i_* are the energy consumption and carbon emissions for energy type *i*. Energy types include electricity, natural gas, liquefied petroleum gas, coal gas, and energy consumption for central heating. *E* is the total energy consumption, *P* is the urban population, *B* is the built-up area, *A* is the area of city, and *I* is the total income. *CC_i_* = *Ci*/*E_i_* is the carbon coefficient for energy *i*, which remains almost unchanged over time. *ES* = *E_i_*/*E* is the energy structure, *EP* = *E*/*P* is the energy consumption per capita, and *PD* = *P*/*B* is the population density in a built-up area. *URS* = *B*/*A* is the urban sprawl effect, indicating the expansion of the built-up area, and *LD* = *A*/*I* is the land-demand effect, reflecting the spatial concentration of urban residents’ income. The smaller the value of *LD*, the higher the spatial concentration of wealth created by residents, and the lower the land demand.

Changes in carbon intensity can be represented by the impacts of these six factors. By reference to these studies [53,54,55], the decomposition of carbon intensity is as follows:

(2) $${}_{△}CI=CI^{t}-CI^{0}={}_{△}CI_{CC}+{}_{△}CI_{ES}+{}_{△}CI_{EP}+{}_{△}CI_{PD}+{}_{△}CI_{URS}+{}_{△}CI_{LD}$$

(3) $${}_{△}CI_{CC}=\sum_{i}\frac{CI_{i}{}^{t}-CI_{i}{}^{0}}{\ln CI_{i}{}^{t}-\ln CI_{i}{}^{0}}\ln\left(\frac{CC_{i}{}^{t}}{CC_{i}{}^{0}}\right)$$

(4) $${}_{△}CI_{ES}=\sum_{i}\frac{CI_{i}{}^{t}-CI_{i}{}^{0}}{\ln CI_{i}{}^{t}-\ln CI_{i}{}^{0}}\ln\left(\frac{ES_{i}{}^{t}}{ES_{i}{}^{0}}\right)$$

(5) $${}_{△}CI_{EP}=\sum_{i}\frac{CI_{i}{}^{t}-CI_{i}{}^{0}}{\ln CI_{i}{}^{t}-\ln CI_{i}{}^{0}}\ln\left(\frac{EP^{t}}{EP^{0}}\right)$$

(6) $${}_{△}CI_{PD}=\sum_{i}\frac{CI_{i}{}^{t}-CI_{i}{}^{0}}{\ln CI_{i}{}^{t}-\ln CI_{i}{}^{0}}\ln\left(\frac{PD^{t}}{PD^{0}}\right)$$

(7) $${}_{△}CI_{URS}=\sum_{i}\frac{CI_{i}{}^{t}-CI_{i}{}^{0}}{\ln CI_{i}{}^{t}-\ln CI_{i}{}^{0}}\ln\left(\frac{URS^{t}}{URS^{0}}\right)$$

(8) $${}_{△}CI_{LD}=\sum_{i}\frac{CI_{i}{}^{t}-CI_{i}{}^{0}}{\ln CI_{i}{}^{t}-\ln CI_{i}{}^{0}}\ln\left(\frac{LD^{t}}{LD^{0}}\right)$$

### 2.2. Data

Income is a better indicator than GDP for representing the beneficial output gained for residents, and therefore, carbon intensity is regarded as the ratio of CO_2_ emissions to income in this study. The data include CO_2_ emissions from urban residential energy consumption, income, urban population, urban built-up area, types of energy consumption, and carbon coefficients. The calculation of CO_2_ emissions is described by Zhao et al. [56], who utilized nighttime light datasets to estimate the spatial and temporal variations in urban residential CO_2_ emissions. Nighttime light datasets have been used as proxies to model socioeconomic activity [57,58]. Due to limitations in statistical data, balanced panel data for 620 county-level cities in 30 provinces (excluding Tibet, Hong Kong, and Macao) for 2000–2015 were used in this study. The relevant data were collected from statistical yearbooks, such as the China City Statistical Yearbooks, the China Statistical Yearbook for Regional Economy, the China Energy Statistical Yearbook, and the provincial statistical yearbooks.

## 3. Results

### 3.1. Temporal and Spatial Characteristics of Carbon Intensity

Increased carbon efficiency plays an important role in reducing CO_2_ emissions. Therefore, it is important to analyze spatial and temporal changes in carbon intensity for carbon-emission reduction and to explore the influencing factors of carbon intensity in the residential sector. In this section, the spatial distribution and temporal trend of carbon intensity are analyzed for 620 county-level cities in China.

**Figure 1:**
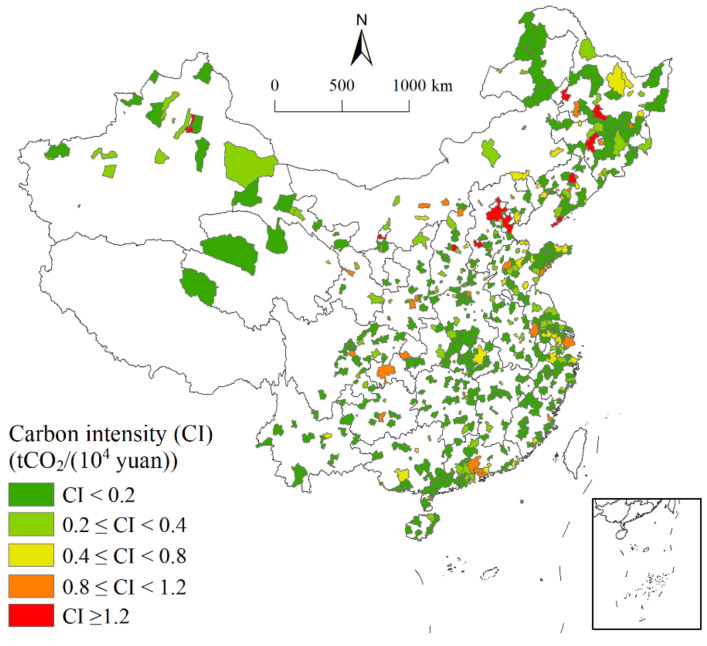
Spatial distribution of carbon intensity during 2001–2015.

Figure 1 depicts the spatial characteristics of carbon intensity. Areas of high carbon intensity are mainly located in Northeast China and the Beijing–Tianjin–Hebei Region. For example, Harbin and Qiqihar in Heilongjiang Province, Shenyang in Liaoning Province, Beijing, and Tianjin all had a carbon intensity of more than 1.2. Areas with a carbon intensity between 0.8 and 1.2 were relatively scattered, including Hohhot, Baotou, and Datong in the northern region, Shanghai and Nanjing in the Yangtze River Delta region, Guangzhou, Shenzhen, and Dongguan in the Pearl River Delta region, Chengdu, and Chongqing. Areas with a carbon intensity ranging from 0.4 to 0.8 were mainly located in the east and northeast, and formed a small-scale agglomeration in the Yangtze River Delta. Areas with a carbon intensity of less than 0.4 were widely distributed.

**Figure 2:**
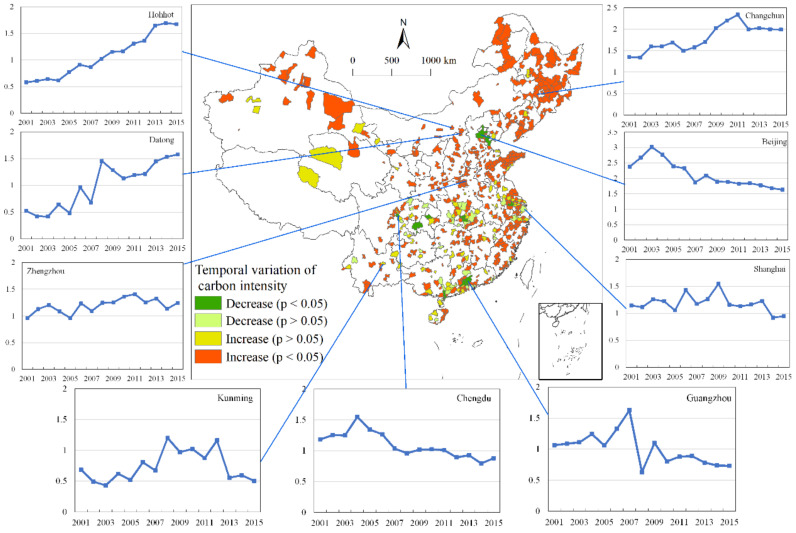
Spatial differentiation of temporal variations of carbon intensity.

A linear regression model was used to test the temporal trends of carbon intensity (Figure 2). The results showed that there is an obvious spatial differentiation in temporal variation. Nine typical cities were selected to present their annual changes in carbon intensity. Cities with high levels of economy, such as Beijing, Shanghai, and Guangzhou, mainly showed a decreasing trend in carbon intensity, which indicated that their carbon efficiency gradually improved and developed towards an energy-intensive type. In addition, cities with a decreasing trend in carbon intensity were mainly located in the south, while cities with an increasing trend were mainly located in the north. This is likely related to the gradual expansion of central-heating coverage [59,60]. With this expansion, the total carbon emissions from residential energy consumption increased somewhat [61]. A comparison of Figure 1 and Figure 2 shows that areas with high carbon intensity mainly showed a downward trend in carbon intensity, while areas with low carbon intensity mainly showed an upward trend in carbon intensity.

**Figure 3:**
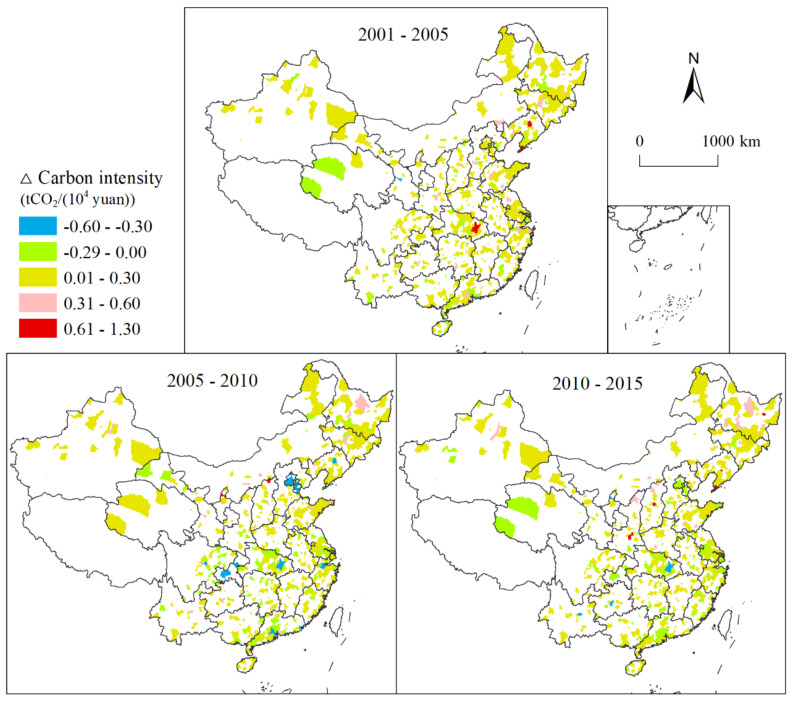
Changes in carbon intensity for the periods 2001–2005, 2005–2010, and 2010–2015.

Three periods were adopted for decomposition analysis: 2001–2005, 2005–2010, and 2010–2015. Changes in carbon intensity for the three periods are shown in Figure 3. Overall, the national carbon intensity tended to decrease with time, and its temporal characteristics exhibited obvious spatial differences. The carbon intensity of Beijing and Tianjin decreased significantly from 2005 to 2010 but increased from 2010 to 2015. The carbon intensity of Heilongjiang had an increasing trend with time. Some cities in central and southern China had a tendency to decrease with time. The carbon intensity of Gansu and Qinghai fluctuated with time. The carbon intensity of Gansu increased from 2005 to 2010, then decreased from 2005 to 2010, before increasing from 2010 to 2015. The trend in carbon intensity in Qinghai was the reverse of that in Gansu.

### 3.2. Decomposition of Carbon Intensity

In order to explore the influences of factors on carbon intensity in depth, the LMDI model was used to decompose the carbon intensity for 620 county-level cities. Carbon intensity was decomposed into six factors: carbon coefficient (CC), energy structure (ES), energy consumption per capita (EP), population density (PD), urban sprawl (URS), and land demand (LD). Since changes in the carbon coefficients were negligible, their effects on carbon intensity were not considered.

#### 3.2.1. Decomposition Analysis on the Provincial Scale

The decomposition results showed that the effects of factors on carbon intensity varied greatly in different spatial units. For macroscopic analysis, the effects of influencing factors on carbon intensity at the provincial level were averaged based on the decomposition results for the county-level cities. The results are displayed in Table 2.

The effect of energy structure (Δ*CI_ES_*) on carbon intensity fluctuated over time in most provinces, and the change in energy structure had the least impact on carbon intensity compared to other factors, which was similar to the results of Jiang [62]. The effects of energy consumption per capita on carbon intensity (Δ*CI_EP_*) were mainly positive in the increase in carbon intensity, which was in accordance with the results of Han et al. [63], and its degree of influence varied in different provinces. In some cities, such as Tianjin, the degree of influence of energy consumption per capita on carbon intensity tended to decrease, while in Shaanxi and Shanghai, its degree of influence exhibited the opposite trend, with an upward tendency. The effects of population density (Δ*CI_PD_*) on carbon intensity presented obvious variations in provinces, and its effects fluctuated over time, mainly contributing to the decrease in carbon intensity; these results are consistent with those found in Song et al. [64]. The land-demand effects (Δ*CI_LD_*) reduced carbon intensity for every province. The carbon intensities of Beijing, Tianjin, Chongqing, and Shanghai were greatly affected by LD; however, they tended to decrease over time, except in Chongqing.

The urban sprawl effects (Δ*CI*_URS_) were positive in stimulating the increase in carbon intensity in all provinces, which was likely because urban sprawl would increase the per capita carbon emissions from energy consumption [44]. The degree of impact of URS on carbon intensity and its temporal trend varied from province to province. In some provinces, such as Beijing, Shanghai, and Guangzhou, the degree of the positive effect of URS on the increase in carbon intensity gradually decreased with time, mainly due to limited urban expansion. Notably, in Shanghai, urban sprawl remained almost zero during 2010–2015. However, in provinces such as Tianjin, Hebei, Ningxia, and Hunan, there was no significant change in the impact of URS on carbon intensity, indicating that their built-up area kept expanding at a certain rate, and the impact on carbon intensity reached a stable level. Some provinces, such as Shanxi, Jiangsu, Guangxi, and Hainan, possessed an increasing influence on carbon intensity with time. Chongqing, Tianjin, Beijing, and Ningxia were the most affected by urban sprawl. The degrees of influence of urban sprawl were seemingly related to the level of economic development.

#### 3.2.2. Decomposition Analysis for Different Types of Cities

• Classification according to the industrial type

There is a big gap in the industrial structure, energy structure, and energy efficiency between traditional and newly industrial regions. We selected some typical cities for the two industrial types displayed in Table 3, and the decomposition results during 2010–2015 are shown.

The effect of EP in traditional industrial cities was slightly higher than that in newly industrial cities. With the aid of technical progress, traditional industrial cities have a lower energy consumption per capita, which contributes to the slower growth of carbon intensity. The urban sprawl effect was slightly lower than that in newly industrial cities; however, the difference was not obvious. The effects of ES, PD, and LD were quite different between the two industrial types. Due to structural adjustments and optimization, the carbon intensity of newly industrial cities was obviously reduced compared with traditional industrial cities. The PD effect reduced the carbon intensity more in newly industrial cities. High-tech industrial zones are usually located in the suburbs, which attracted people away from urban areas, and the population density decreased in built-up areas. In the meanwhile, the total energy consumption and carbon emissions decreased. Furthermore, thanks to the improvement of technological level, the carbon intensity declined in newly industrial cities. The LD effect was bigger in newly industrial cities than that in traditional cities. The development of high-tech zones had a positive effect on urban land-use efficiency [65], usually with a high level of innovation and promotion of the economic development.

• Classification according to the administrative and economic level

The 620 county-level cities were divided into three categories: municipal districts of provincial capital cities (Type A cities), municipal districts of prefecture-level cities, except for Type A cities (Type B cities), and county-level cities, except for Type A and B cities (Type C cities). The results of decomposition for the different types of cities are shown in Table 4.

The changes in ES were related to the increase in carbon intensity for the periods 2001–2005 and 2005–2010. In Type A and B cities, the degree of the effect of ES on carbon intensity showed a downward trend, while it showed an upward trend in Type C cities. During the period 2010–2015, the changes in ES were related to the decrease in carbon intensity for the three types of cities. Even so, the effect of ES on carbon intensity was small compared with those of other factors. In addition, ES had the most obvious effect on the carbon intensity of Type A cities. There was no significant difference in the effect of ES on carbon intensity between Type B and C cities.

Changes in EP had a positive and relatively large effect on the increase in carbon intensity. The degree of influence of EP on carbon intensity first increased and then decreased with time. The influence of EP on carbon intensity was greatest in Type A cities, followed by Type B cities, and then in Type C cities.

From 2001 to 2015, the influence of PD on carbon intensity fluctuated. The degree of the effect of PD on carbon intensity was relatively small—only slightly greater than that of ES. PD was more influential in Type A cities than in other types of cities.

URS was the most stable factor, and there was no obvious change in the degree of influence of URS on carbon intensity with time. Notably, the effect of URS on carbon intensity in Type A cities was significantly greater than in Type B and C cities, indicating that URS played a greater role in increasing carbon emissions in provincial capitals.

The LD factor showed a negative effect on the increase in carbon intensity, with the degree of the effect first increasing and then decreasing with time. The degree of the influence of LD on carbon intensity in Type A cities was obviously greater than in Type B cities and was smallest in Type C cities.

In short, the factors’ effects exhibited clear temporal variation. The most influential factors were urban sprawl, land demand, and energy consumption per capita—urban sprawl is closely related to land-use change. In addition, the factors showed the greatest influence in the municipal districts of provincial capital cities, mainly due to their high carbon intensity.

• Classification according to the spatial location

There are large climatic differences between the northern and southern regions. Because the northern regions have a lower air temperature, they consume more energy for heating in the winter. In the summer, the southern regions have a higher air temperature and spend more energy on cooling.

As shown in Table 5, the change in carbon intensity was greater in the northern cities than in the southern cities. There were two reasons for this result. One was that carbon intensity in northern cities was higher than that in southern cities, and thus provided plenty of room for the carbon intensity to decrease. The other was that the change in carbon intensity was also larger than that in the southern cities. Another characteristic was that the gap in the changes in carbon intensity between the northern and southern cities was much greater in Type A cities than in Type B cities. This finding further confirmed that changes in carbon intensity in Type A cities should be paid more attention.

#### 3.2.3. Decomposition Analysis for Capital Cities

Since the factors had the greatest impact on carbon intensity in the municipal districts of provincial capital cities, which are energy-intensive and densely populated areas, it is necessary to further explore the regularity and features of the influencing factors for carbon intensity in these cities. The results of the decomposition for the municipal districts of provincial capital cities are displayed in Table 6.

Changes in ES had the least impact on carbon intensity. During the study period, changes in ES first exhibited a positive effect on the increase in carbon intensity, and then showed a negative effect in some provincial capitals, such as Tianjin, Shijiazhuang, Taiyuan, Hohhot, Shanghai, Nanjing, Hefei, Wuhan, Chongqing, Xi’an, and Lanzhou. However, in Beijing, Shenyang, and Fuzhou, the temporal trend of the effect of ES was the opposite. The impacts of ES remained unchanged in Hangzhou, Nanchang, Changsha, Nanning, Chengdu, and Urumchi, among which only Urumchi showed a constantly restraining effect on the increase in carbon intensity.

With regard to the impact of EP, there were 12 cities with constantly positive effects on the increase in carbon intensity. The effect of EP in Shenyang during 2001–2005 was largest, and in Taiyuan and Xi’an during 2010–2015. In terms of the temporal variation, there was no significant change in the impact of EP on carbon intensity in Beijing. The impact of EP on carbon intensity in Tianjin, Nanchang, and Changsha tended to decrease, while in Shijiazhuang, Harbin, Fuzhou, and Jinan, it tended to increase. Most cities showed a fluctuating growth trend in the effect of EP on carbon intensity. For example, Chongqing showed an effect of increasing carbon intensity during 2001–2005 and 2010–2015, as well as an effect of reducing carbon intensity during 2005–2010. The degree of the effect of EP in Hefei first increased, and then decreased. Guangzhou showed a positive effect on the increase in carbon intensity during 2001–2005 and a negative effect during 2005–2015.

There were spatiotemporal differences in the influence of PD on carbon intensity. The effects of PD on reducing carbon intensity during 2001–2005 were relatively greater than the effects on increasing carbon intensity, such as in Chongqing, Shenyang, Harbin, Nanjing, and Zhengzhou. During 2005–2010, the effects of increasing carbon intensity were relatively greater, such as in Zhengzhou, which had the largest degree of effect, followed by Hohhot, Harbin, and Beijing. Changchun and Wuhan exhibited relatively large effects of reducing carbon intensity. During 2010–2015, the overwhelming majority of cities showed a decreasing trend in the degree of influence of PD, except for Hangzhou, which showed an increasing trend.

The effects of URS on increasing carbon intensity showed slight regional differences. The greatest promotive effect of URS on carbon intensity was found in Beijing during 2001–2005, while during 2005–2010, the biggest URS effect was identified in Changchun and Urumchi. There were no obvious regional differences in the positive effects of URS on the increase in carbon intensity during 2010–2015. In terms of temporal variations, Tianjin, Nanchang, Jinan, Changsha, Nanning, and Haikou showed stable effects of URS on carbon intensity. The effects of URS in Taiyuan, Hohhot, Xi’an, Lanzhou, and Xining had a steady upward trend. However, the effects of URS in most cities—Shijiazhuang, Nanjing, Hangzhou, Hefei, Zhengzhou, and Beijing—had a steady downward trend. Shenyang, Changchun, Harbin, and Chengdu had a fluctuating downward trend for the effect of URS.

LD had a negative effect on the increase in carbon intensity in all provincial capitals, and the degree of the effect mainly had an increasing trend, such as in Shijiazhuang, Taiyuan, Zhengzhou, and Xi’an. A few large cities showed a decreasing trend in the degree of the effect of LD, such as Beijing, Shanghai, and Guangzhou. The effects of LD in Nanjing, Hangzhou, Hefei, and Haikou were stable over time.

## 4. Discussion

Carbon intensity is an important indicator of carbon efficiency, and improvements in carbon efficiency and technology play an essential role in carbon reduction [56]. In our study, a similar conclusion was drawn regarding such improvements. Therefore, it is crucial to increase carbon efficiency in order to reduce carbon emissions and, thus, to mitigate global warming.

Three factors influenced carbon intensity the most: the energy consumption per capita effect, the urban sprawl effect, and the land-demand effect.

Energy consumption per capita mainly showed an effect of increasing carbon intensity, which could be explained by the following: Firstly, the ownership of appliances increased rapidly, including per-household ownership [66]—a major driving force in electricity consumption and carbon emissions. Secondly, with improvements in living standards, residents pursue a more comfortable life, resulting in, for example, a rise in domestic hot water and air-conditioning use [67]. Thirdly, due to the demolition and renovation of old residential communities, the central heating system was expanded, resulting in an increase in energy consumption and pollutant emissions.

The urban sprawl effect, the major concern in this study, is related to changes in land use in urban areas. With the expansion of built-up areas, various land-use types were converted into construction land, thus changing urban morphology and making cities less compact. In this study, the expansion of built-up areas had a significantly positive effect on the increase in carbon intensity. Likewise, Wang et al. [68] came to a similar conclusion—that urban sprawl is negatively correlated with carbon-emission efficiency, which is usually the reciprocal of carbon intensity. There are several reasons for this. Firstly, dwellers reside in a greater degree of dispersion compared with those living in a compact city, which results in an increase in heat loss from long heating pipes [27]. Secondly, land-use change decreases green-land cover, and carbon absorption declines [69]. In addition, many studies have shown that urban size is positively related to carbon emissions [70,71].

The land-demand effect reduced the carbon intensity, indicating that the wealth created by laborers per unit of land area showed an increasing trend. The land-demand effect was related to the economic level of the city, and the degree of its effect was relatively strong for large cities and provincial capitals. The main reason for this result was that the economic levels and economic efficiencies of large cities are relatively high, and the added value created per unit of input is greater, thus substantially reducing land demand.

Additionally, population density mainly showed an effect of reducing carbon intensity, which is essentially consistent with the conclusion of Huo et al. [72]; the authors argued that a low population density leads to a low final energy demand, resulting in a low carbon intensity. Similarly, Song et al. [64] pointed out that population concentration had an inhibitory effect on carbon-emission intensity in China’s Bohai Economic Rim. In our study, the population density was related to city size. The degree of the effect of population density on decreasing carbon intensity had a slightly enhanced trend in Type B and Type C cities, meaning that there was a steeply decreasing trend in population density in small-to-medium cities. For provincial capitals, the degree of impact of population density mainly showed a weakened trend, especially in big cities, such as Beijing, Shanghai, and Guangzhou, where the changes in population density were related to the increase in carbon intensity. This is likely because large cities provide more jobs and have higher welfare benefits than small cities, thus proving more attractive to people and encouraging population inflow. Meanwhile, the expansion of built-up areas in large cities tends to be stable, increasing the population density in those areas.

## 5. Conclusions

In this study, the relationship between carbon emissions and carbon intensity was examined. We explored the causes of changes in carbon intensity using balanced panel data from 620 county-level cities in 30 provinces (excluding Tibet, Hong Kong, and Macao) and the LMDI model. Firstly, the spatial and temporal characteristics of carbon intensity were identified. Secondly, we employed the LMDI model to decompose the carbon intensity into six factors, including the carbon coefficient, energy structure, energy consumption per capita, population density, urban sprawl, and land demand. Urban sprawl is the ratio of the built-up area to a city’s size, which is accompanied by land-use change and reflects the influence of human activity on land use.

The analysis showed that there was obvious spatial heterogeneity in carbon-emission intensity and its temporal trends. Clearly, large cities, such as Beijing, Tianjin, and Shanghai, had a relatively high carbon intensity, which, however, showed a downward trend. In addition, many cities in southern China also showed an overall downward trend in carbon-emission intensity. The decomposition analysis showed that the energy consumption per capita, urban sprawl, and land demand influenced carbon intensity the most. Energy consumption per capita mainly had a positive effect on the increase in carbon intensity—its degree of influence was high in the municipal districts of provincial capital cities, but relatively low in other types of cities. The urban sprawl effect increased carbon intensity in most cities, and the degree of its effect was significantly greater in big cities compared with small-to-medium cities. However, as urban expansion tended to flatten out, the urban sprawl effect decreased, especially in Shanghai. The land-demand effect exerted a negative effect on the increase in carbon intensity, and its degree of influence was clearly strong in big cities.

Based on these results, some policy suggestions are provided for reducing carbon emissions and carbon intensity. Firstly, lowering the energy consumption per capita is one of the most effective ways to reduce carbon intensity. It is essential to further improve residents’ awareness of energy saving, guiding them to conserve energy and, thus, reduce carbon emissions. Central heating in winter constitutes a large proportion of urban residential energy consumption, and there is great potential for energy conservation. However, residents rarely intentionally decrease heat input, even if it is warm enough. Therefore, some measures should be taken. For example, the charges for central heating should be based on both the heating area and the input flow. In addition, step pricing can be used to regulate the consumption of household electricity. Secondly, the positive impact of urban expansion on the increase in carbon intensity needs to be acknowledged, especially in big cities, which should be attributed to the rapid land urbanization. For most cities, large-scale land expansion is underway, and urban expansion will most likely increase carbon intensity. That is to say, the land utilization efficiency should be increased by optimizing the land-use structure for these big cities to offset the effect of urban sprawl. Thirdly, regional differences in the effects of factors on carbon intensity must be considered by decision-makers. The developmental speed of each city is not the same, and neither is the developmental stage. Therefore, the effects of differences in influencing factors are relatively large, and mitigation countermeasures according to local needs must be implemented.

## Figures and Tables

**Table 1:** Representative studies related to residential carbon emissions and carbon intensity.

Author(s)	Study Period	Research Object	Method(s)	Study Scale
Greening et al., (2001) [32]	1970–1993	Carbon intensity of residential end-uses in 10 OECD countries	Adaptive weighting Divisia index decomposition method	National level
Zhang. et al., (2009) [43]	1991–2006	Energy-related CO_2_ emissions in China	Complete decomposition method	National level
Liu et al., (2015) [44]	1996–2012	Carbon intensity in China’s 12 industrial sectors	LMDI	National level
Cheng et al., (2018) [45]	1998–2014	Carbon intensity in China’s 30 provinces	Spatial econometric model	Provincial level
Liu et al., (2019) [33]	1995–2010	China’s household carbon intensity	LMDI model and STIRPAT model	National level
Liu et al., (2019) [34]	2002–2012	Carbon emissions of urban households in China	LMDI	National level
Yuan et al., (2019) [46]	2012	Household carbon emissions in China’ 30 provinces	Spatial decomposition analysis	Provincial level
Fan and Fang (2020) [47]	2002–2012	Residential CO_2_ emissions in Qinghai	Structural decomposition analysis	Provincial level
Tomas B. (2020) [48]	2004–2016	CO_2_ emissions in the residential sector in Lithuania	Index decomposition analysis	National level
Meng et al., (2021) [49]	2005–2015	CO_2_ emission reduction in residential sectors in China’ 286 cities	Laspeyres index decomposition method	Provincial and city levels

**Table 2:** Results of the decomposition analysis of carbon intensity on the provincial scale (tCO_2_/(10^4^ yuan)).

Provinces	2010–2015					2005–2010					2001–2005				
	Δ*CI_ES_*	Δ*CI_EP_*	Δ*CI_PD_*	Δ*CI_URS_*	Δ*CI_LD_*	Δ*CI_ES_*	Δ*CI_EP_*	Δ*CI_PD_*	Δ*CI_URS_*	Δ*CI_LD_*	Δ*CI_ES_*	Δ*CI_EP_*	Δ*CI_PD_*	Δ*CI_URS_*	Δ*CI_LD_*
Beijing	0.014	0.204	0.051	0.162	−0.642	0.060	0.201	0.420	0.154	−1.182	−0.087	0.383	0.280	1.132	−1.699
Tianjin	−0.050	0.072	0.140	0.361	−0.652	0.010	0.460	−0.155	0.394	−0.961	0.118	0.629	−0.294	0.381	−0.768
Hebei	−0.019	0.080	−0.022	0.057	−0.037	−0.002	0.080	0.031	0.056	−0.032	0.015	0.034	−0.003	0.052	−0.021
Shanxi	−0.008	0.180	−0.037	0.082	−0.025	0.010	0.102	0.042	0.048	−0.026	0.012	0.065	0.000	0.017	−0.018
Inner Mongolia	−0.021	0.177	−0.047	0.061	−0.038	0.007	0.054	0.073	0.062	−0.053	0.002	0.093	−0.025	0.037	−0.024
Liaoning	0.008	0.131	−0.027	0.065	−0.012	−0.004	0.101	0.011	0.101	−0.106	−0.012	0.188	−0.019	0.041	−0.047
Jilin	−0.007	0.103	−0.006	0.033	−0.032	−0.006	0.080	−0.007	0.074	−0.046	−0.009	0.082	−0.018	0.041	−0.016
Heilongjiang	−0.010	0.184	−0.017	0.032	−0.019	−0.012	0.091	0.009	0.038	−0.046	0.002	0.062	−0.025	0.042	−0.029
Shanghai	−0.052	0.104	0.047	0.000	−0.210	0.177	0.037	0.312	0.231	−0.531	0.086	−0.074	−0.018	0.506	−0.618
Jiangsu	−0.003	0.020	−0.001	0.045	−0.047	0.009	0.029	−0.023	0.058	−0.049	0.026	0.005	0.000	0.062	−0.035
Zhejiang	0.010	0.006	0.002	0.032	−0.029	0.031	−0.080	0.087	0.038	−0.032	0.028	0.034	−0.017	0.046	−0.020
Anhui	−0.018	0.036	−0.011	0.037	−0.020	0.001	0.008	0.025	0.030	−0.024	0.022	0.026	−0.022	0.043	−0.011
Fujian	0.006	0.015	−0.014	0.046	−0.030	0.018	0.025	−0.030	0.073	−0.034	0.020	−0.016	0.020	0.043	−0.011
Jiangxi	−0.002	0.021	−0.006	0.038	−0.018	0.008	−0.001	0.000	0.044	−0.017	0.021	0.037	−0.018	0.031	−0.007
Shandong	−0.014	0.084	−0.020	0.071	−0.044	0.011	0.068	0.012	0.074	−0.050	−0.002	0.072	−0.032	0.075	−0.027
Henan	−0.007	0.071	−0.013	0.037	−0.022	0.006	0.029	0.039	0.038	−0.019	0.000	0.024	−0.021	0.038	−0.009
Hubei	−0.004	0.020	−0.014	0.036	−0.019	−0.006	0.004	0.001	0.030	−0.021	0.022	0.041	0.002	0.001	−0.010
Hunan	−0.009	0.046	0.004	0.026	−0.026	0.007	−0.001	0.013	0.042	−0.029	0.016	0.028	−0.002	0.022	−0.009
Guangdong	0.004	0.027	−0.014	0.053	−0.042	0.032	−0.104	0.081	0.062	−0.055	0.012	0.036	0.000	0.081	−0.059
Guangxi	0.009	0.013	−0.013	0.038	−0.020	0.013	−0.008	0.029	0.024	−0.018	0.005	0.013	0.000	0.018	−0.008
Hainan	0.009	0.017	−0.021	0.043	−0.009	−0.001	−0.001	0.031	0.011	−0.006	0.008	−0.003	−0.007	0.021	−0.003
Chongqing	−0.020	0.214	−0.201	0.447	−0.610	0.035	−0.462	0.343	0.481	−0.903	0.076	0.364	−0.643	0.856	−0.482
Sichuan	0.001	0.037	−0.014	0.061	−0.030	−0.005	−0.021	0.022	0.029	−0.028	0.032	0.030	−0.013	0.043	−0.014
Guizhou	−0.028	−0.003	−0.034	0.052	−0.022	0.016	0.036	0.036	0.031	−0.015	0.003	0.044	−0.014	0.018	−0.005
Yunnan	0.000	−0.002	−0.010	0.024	−0.014	0.020	−0.020	0.037	0.038	−0.014	0.006	0.004	−0.024	0.024	−0.006
Shaanxi	−0.009	0.136	−0.041	0.075	−0.047	0.002	0.037	0.013	0.063	−0.041	0.006	0.069	−0.015	0.026	−0.020
Gansu	−0.003	0.051	−0.025	0.064	−0.020	−0.015	0.067	0.013	0.046	−0.014	0.015	0.002	0.003	0.021	−0.011
Qinghai	−0.028	0.076	−0.008	0.054	−0.020	0.014	0.025	0.052	0.010	−0.017	−0.019	0.065	0.002	0.008	−0.006
Ningxia	0.094	−0.162	−0.035	0.124	−0.034	−0.095	0.313	0.044	0.112	−0.042	−0.014	0.103	−0.052	0.101	−0.015
Xinjiang	−0.013	0.129	−0.047	0.081	−0.036	−0.001	0.029	0.016	0.088	−0.033	−0.008	0.063	0.000	0.016	−0.016

**Table 3:** Decomposition results for different types of industrial cities during 2010–2015 (tCO_2_/(10^4^ yuan)).

Factors	Traditional Industrial Cities	Newly Industrial Cities
Cities	Harbin, Yichun, Changchun, Siping, Shenyang, Pingdingshan, Zhuzhou, Xiangtan, Shaoxing, Liuzhou, Baise	Shenzhen, Guangzhou, Hangzhou, Nanjing, Wuhan, Xi’an, Suzhou, Changsha, Chengdu, Qiangdao, Xiamen, Wuxi, Hefei, Jinan, Ningbo
Δ*CI_ES_*	−0.009	−0.029
Δ*CI_EP_*	0.183	0.170
Δ*CI_PD_*	−0.013	−0.033
Δ*CI_URS_*	0.130	0.200
Δ*CI_LD_*	−0.174	−0.287

**Table 4:** Results of the decomposition analysis of carbon intensity for different types of cities (tCO_2_/(10^4^ yuan)).

Period	Factors	Type A Cities	Type B Cities	Type C Cities
2010–2015	Δ*CI_ES_*	−0.0373	−0.0034	−0.0030
	Δ*CI_EP_*	0.3375	0.0883	0.0325
	Δ*CI_PD_*	−0.0438	−0.0239	−0.0103
	Δ*CI_URS_*	0.2691	0.0711	0.0234
	Δ*CI_LD_*	−0.3734	−0.0331	−0.0068
2005–2010	Δ*CI_ES_*	0.0326	0.0040	0.0053
	Δ*CI_EP_*	0.1007	0.0335	0.0171
	Δ*CI_PD_*	0.0322	0.0272	0.0235
	Δ*CI_URS_*	0.3591	0.0734	0.0203
	Δ*CI_LD_*	−0.4555	−0.0484	−0.0091
2001–2005	Δ*CI_ES_*	0.0429	0.0212	0.0017
	Δ*CI_EP_*	0.2623	0.0640	0.0241
	Δ*CI_PD_*	−0.1345	−0.0107	−0.0043
	Δ*CI_URS_*	0.3856	0.0576	0.0128
	Δ*CI_LD_*	−0.3269	−0.0267	−0.0043

**Table 5:** Decomposition results for the northern and southern cities during 2010–2015 (tCO_2_/(10^4^ yuan)).

Types	Administrative Level	Δ*CI_ES_*	Δ*CI_EP_*	Δ*CI_PD_*	Δ*CI_URS_*	Δ*CI_LD_*
Northern cities	Type A cities	−0.079	0.576	−0.088	0.348	−0.436
	Type B cities	−0.006	0.141	−0.036	0.084	−0.033
Southern cities	Type A cities	0.007	0.080	0.004	0.184	−0.306
	Type B cities	−0.001	0.039	−0.013	0.059	−0.033

**Table 6:** Results of the decomposition analysis for capital cities (tCO_2_/(10^4^ yuan)).

Cities	2010–2015					2005–2010					2001–2005				
	Δ*CI_ES_*	Δ*CI_EP_*	Δ*CI_PD_*	Δ*CI_URS_*	Δ*CI_LD_*	Δ*CI_ES_*	Δ*CI_EP_*	Δ*CI_PD_*	Δ*CI_URS_*	Δ*CI_LD_*	Δ*CI_ES_*	Δ*CI_EP_*	Δ*CI_PD_*	Δ*CI_URS_*	Δ*CI_LD_*
Beijing	0.014	0.204	0.051	0.162	−0.642	0.060	0.201	0.420	0.154	−1.182	−0.087	0.383	0.279	1.132	−1.699
Tianjin	−0.050	0.072	0.140	0.361	−0.652	0.010	0.460	−0.155	0.394	−0.961	0.118	0.629	−0.294	0.381	−0.768
Shijiazhuang	−0.153	0.508	−0.017	0.225	−0.581	−0.030	0.359	−0.085	0.292	−0.244	0.060	0.225	−0.090	0.473	−0.151
Taiyuan	−0.268	2.521	−0.314	0.698	−0.389	0.247	−0.353	0.148	0.289	−0.344	0.007	0.621	0.030	0.129	−0.284
Hohhot	−0.089	0.566	−0.280	0.465	−0.273	0.140	−0.052	0.448	0.136	−0.223	0.028	0.314	−0.111	0.118	−0.156
Shenyang	0.018	0.404	−0.129	0.332	−0.438	0.152	0.241	−0.350	0.764	−1.260	−0.296	2.116	−0.523	0.589	−0.577
Changchun	−0.146	0.071	0.133	0.344	−0.566	0.029	0.698	−0.578	1.036	−0.571	−0.124	0.615	−0.335	0.489	−0.290
Harbin	0.110	0.747	−0.049	0.271	−0.566	−0.054	0.135	0.431	0.234	−0.716	0.031	0.094	−0.504	0.827	−0.609
Shanghai	−0.052	0.104	0.047	0.000	−0.210	0.177	0.037	0.312	0.231	−0.531	0.086	−0.074	−0.018	0.506	−0.618
Nanjing	−0.031	0.084	0.016	0.147	−0.374	0.060	0.281	−0.001	0.166	−0.346	0.171	−0.113	−0.443	0.769	−0.362
Hangzhou	0.012	0.014	0.221	0.104	−0.275	0.012	−0.253	0.102	0.187	−0.296	0.149	0.285	0.027	0.232	−0.235
Hefei	−0.095	0.005	0.085	0.196	−0.227	−0.134	0.111	0.092	0.268	−0.297	0.186	0.058	−0.170	0.354	−0.135
Fuzhou	0.054	0.279	−0.001	0.164	−0.269	0.155	0.178	−0.193	0.311	−0.270	−0.008	0.001	−0.043	0.387	−0.073
Nanchang	0.018	0.005	−0.105	0.246	−0.190	0.036	0.048	−0.111	0.281	−0.192	0.162	0.172	−0.157	0.257	−0.100
Jinan	−0.138	0.579	−0.058	0.137	−0.242	0.058	0.405	−0.350	0.385	−0.374	−0.005	0.195	0.079	0.283	−0.195
Zhengzhou	−0.122	0.301	0.038	0.266	−0.475	0.139	−0.302	0.814	0.308	−0.357	−0.084	0.107	−0.441	0.583	−0.166
Wuhan	−0.043	−0.034	−0.123	0.124	−0.322	−0.253	−0.095	−0.463	0.858	−0.426	0.471	0.310	0.142	0.048	−0.246
Changsha	0.002	0.066	0.153	0.132	−0.395	0.092	0.182	−0.264	0.669	−0.509	0.026	0.301	0.054	0.125	−0.191
Guangzhou	0.057	−0.022	0.123	0.189	−0.360	−0.057	−0.140	0.312	0.231	−0.470	0.042	0.292	−0.295	0.458	−0.505
Nanning	0.069	0.095	−0.010	0.160	−0.154	0.020	0.167	−0.014	0.123	−0.151	0.035	−0.067	0.073	0.163	−0.066
Haikou	0.080	0.001	−0.036	0.159	−0.055	−0.063	0.120	0.090	0.001	−0.030	0.037	−0.105	−0.074	0.160	−0.022
Chongqing	−0.020	0.214	−0.201	0.447	−0.610	0.035	−0.462	0.343	0.481	−0.903	0.076	0.364	−0.643	0.856	−0.482
Chengdu	0.043	0.234	−0.116	0.329	−0.538	0.215	0.004	0.059	0.166	−0.583	0.063	−0.087	−0.155	0.682	−0.344
Xi’an	−0.121	1.122	−0.292	0.584	−0.463	−0.018	0.223	−0.152	0.373	−0.432	0.004	0.493	−0.099	0.200	−0.214
Lanzhou	−0.015	0.063	−0.221	0.466	−0.243	−0.193	0.677	−0.140	0.303	−0.126	0.150	−0.594	0.008	0.115	−0.122
Xining	−0.125	0.279	−0.023	0.143	−0.059	0.069	−0.022	0.131	0.019	−0.046	−0.078	0.223	0.001	0.020	−0.016
Urumchi	−0.015	0.629	−0.214	0.418	−0.516	−0.024	−0.127	0.024	1.036	−0.456	−0.061	0.324	0.070	0.075	−0.203

## Data Availability

The data presented in this study are available on reasonable request from the corresponding author.
